# Chimeric antigen receptor modified hematopoietic stem cells (CAR-HSCs) arm all immune forces for anti-tumor in mice

**DOI:** 10.1186/s40164-025-00715-7

**Published:** 2025-10-22

**Authors:** Tao Wang, Ping Liu, Dongliang Zhang, Zhiqiang Song, Mingyang Yu, Dongge Feng, Xuejun Yu, Na Liu, Gusheng Tang, Jianmin Yang

**Affiliations:** 1 Department of Hematology, Institute of Hematology, Changhai Hospital, Naval Medical University, 168 Changhai Road, Shanghai, 200433 China; 2 Huadao Biopharma (Shanghai) Limited Corporation, Shanghai, China; 3 Department of Joint Bone Disease Surgery, Changhai Hospital, Naval Medical University, Shanghai, 200433 China; 4 Department of Orthopaedics, the 922nd Hospital of the Joint Service Support Force of the PLA, Hengyang, 421002 China

**Keywords:** Tumor, Chimeric antigen receptor, Hematopoietic stem cell, Transplantation, Tumor microenvironment

## Abstract

Chimeric antigen receptor T cell (CAR-T) is the main salvage therapy for relapsed or refractory large B-cell lymphoma (r/r LBCL). However, over 50% of patients relapse after CAR-T therapy. In this work, we transduced the CAR gene into hematopoietic stem cells (HSCs) using a lentiviral vector. Chimeric antigen receptor-modified HSC (CAR-HSCs) were transplanted into mice after lethal irradiation. CAR gene transduction did not compromise the ability of HSCs to expand, self-renew, or reconstitute. CAR was expressed on T cells, natural killer cells, B cells, monocytes, and neutrophils in the peripheral blood. CAR-HSCs transplantation significantly reduced CD19^+^ tumor burden and prolonged the survival of mice with preclinical tumor without severe toxicity. CAR-HSCs also differentiated into different CAR-expressing immune cells that reshaped the tumor microenvironment by increasing the proportion of antitumor cells (like CD8^+^ T cells) and the antitumor response, and by decreasing immunosuppressive cells, such as tumor-associated macrophage subtype 2. This study demonstrated a preclinical proof-of-principle for CAR-HSCs therapy in r/r LBCL, suggesting an opportunity for its clinical translation.

**Supplementary Information:**

The online version contains supplementary material available at 10.1186/s40164-025-00715-7.

## Dear editor

Anti-CD19 chimeric antigen receptor T cell (CAR-T) has shown remarkable success in treating relapsed or refractory large B-cell lymphoma (r/r LBCL), with a complete remission rate over 60% [1, 2]. However, the 5-year survival after CAR-T therapy is only 40% [3]. The main reasons for relapse or progression after CAR-T therapy are the limited lifespan of CAR-T cells [4], CD19 antigen escape [5], and the immunosuppressive tumor microenvironment (TME) [6–8].

We previously reported that anti-CD19 CAR-T sequentially followed by autologous hematopoietic stem cell transplantation (ASCT) improved 3-year progression-free survival in r/r LBCL patients compared to ASCT alone [9]. However, the lifespan of CAR-T cells is limited. The ability of hematopoietic stem cells (HSCs) to self-renew and multiple cell lineage differentiation make them ideal for CAR-T cell-based immunotherapy [10, 11]. In addition, CAR-NK [12] and CAR-macrophage (CAR-M) [13] have demonstrated antitumor activity. Viral and non-viral vector-based gene editing of HSC and ASCT has been used in clinical practice, mainly in monogenic disorder diseases [14]. In this study, we employed HSCs to express CAR, constructed CAR-modified hematopoietic stem cells (CAR-HSCs), and transplanted them into a murine model to clarify the characteristics of hematopoietic reconstitution and differentiation, and assess CAR-HSCs antitumor effects and TME reshaping (Fig. S1).

First, we transfected HSCs with a CAR-encoding lentiviral vector, with a transfection rate of 20.05% (Fig. S2A). The colony-forming unit analysis of CAR-HSCs showed no significant difference compared with unmodified HSCs (CTRL) (Fig. S2B). We used B6-CD45.1 as donor and B6-CD45.2 as recipient mice to mimic ASCT and analyze the hematopoietic reconstitution of CAR-HSCs [15]. 28 days after ASCT, the CAR-HSC group and the CTRL group demonstrated comparable chimeric rates (CD45.1^+^/CD45^+^), total white cell counts, and T and NK cell proportions and count (Fig. S3A-E). The proportion and count of B cells in the CAR-HSC group was lower than in the CTRL group (*P* = 0.001) (Fig. S3F). The proportion and count of neutrophils (*P* = 0.001) and monocytes (*P* = 0.036) in the CAR-HSC group was higher than in the CTRL group (Fig. S3G-H). The mean expression of CAR on immune cells was 12.93% (Fig. S3I). The CAR expressing cells including 7.23% of B cells, 6.00% of T cells, 15.15% of NK cells, 53.79% of neutrophils, and 17.75% of monocytes (Fig. S3J-K). These results indicate that the CAR-encoding lentiviral vector transfection did not compromise the ability of HSCs to differentiate and reconstitute.

**Fig. 1:**
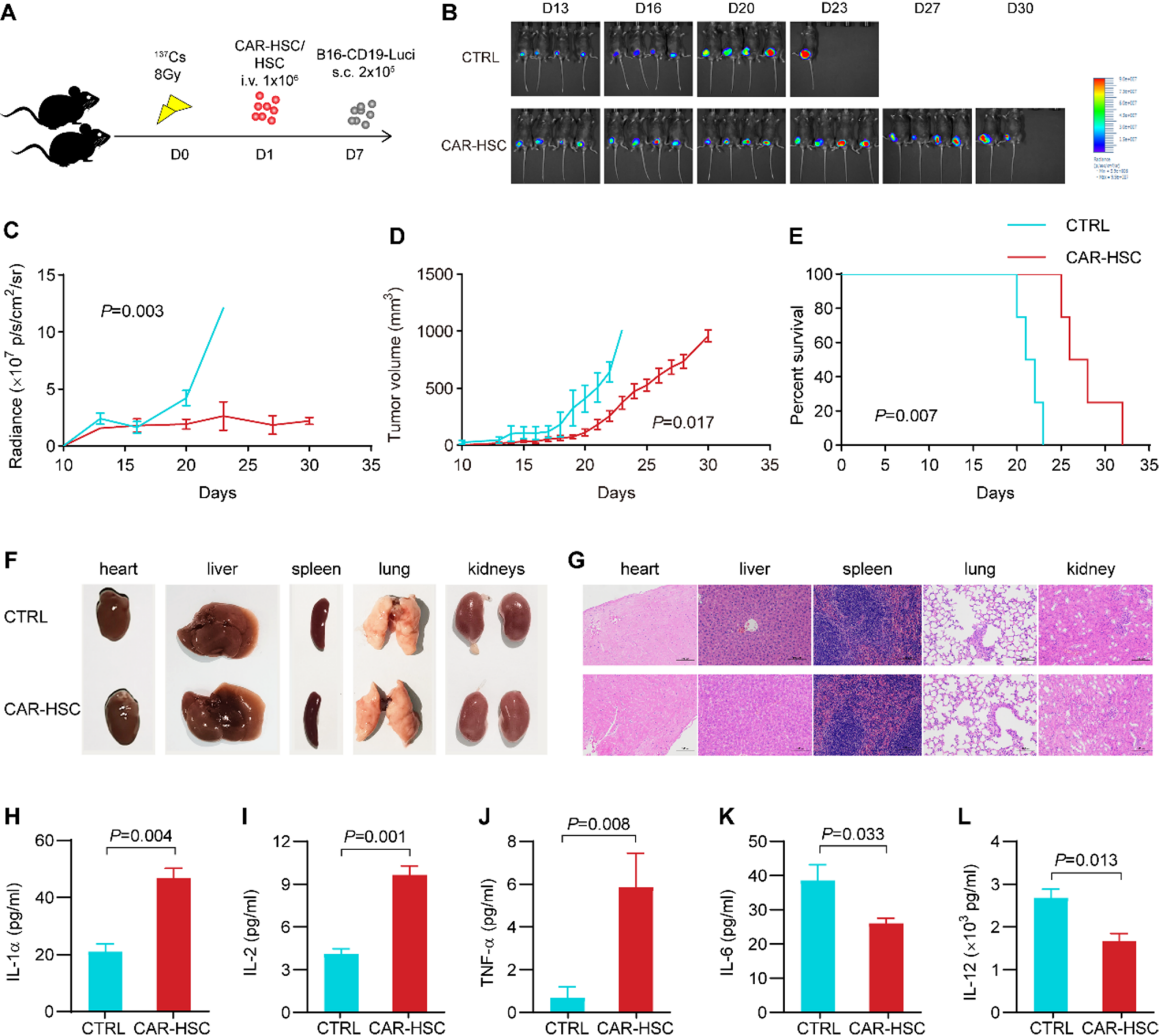
Efficacy and safety of CAR-HSCs transplantation on CD19^+^ tumor cells. **A** Schematic illustration of CAR-HSCs transplantation and tumor model construction. **B** Imaging of B16-CD19-Luc tumor bearing C57/BL6 mice treated with CAR-HSCs or HSCs (CTRL) transplantation. **C** Quantification of B16-CD19-Luc tumor burden (*n* = 5). **D** Tumor volume of B16-CD19-Luc tumor bearing C57/BL6 mice (*n* = 5). **E** Survival of B16-CD19-Luc tumor bearing mice treated with CAR-HSC or HSC (CTRL) transplantation (*n* = 5). **F**–**G** Shape and hematoxylin and eosin staining of murine heart, liver, spleen, lung and kidneys treated with CAR-HSCs or HSCs transplantation. **H**–**L** Cytokines in the serum of mice treated with CAR-HSCs or HSCs transplantation on 28 day post-transplantation (*n* = 3)

Next, we assessed the anti-tumor effect of CAR-HSCs for CD19^+^ tumors in vivo (Fig. 1A). The results revealed that tumor growth in the mice transplanted with CAR-HSCs was slower than in the CTRL group (*P* = 0.002) (Fig. 1B-D). CAR-HSCs transplantation prolonged the survival of the mice (*P* = 0.007) (Fig. 1E). As for toxicities, no differences in morphology or histological structure were observed in main organs between the two groups (Fig. 1F-G). Twenty-three serum cytokines were evaluated on 28 day post-transpalntation, to assess the risk of cytokine release syndrome (CRS) (Fig. S4). The results demonstrated that the concentrations of interleukin (IL)-1α (*P* = 0.004), IL-2 (*P* = 0.001), and tumor necrosis factor (TNF)-α (*P* = 0.008) were increased, whereas the levels of IL-6 (*P* = 0.033) and IL-12 (*P* = 0.013) were decreased in the CAR-HSC group compared to the CTRL group (Fig. 1H-L). No mice died of CRS or other toxicity.

**Fig. 2:**
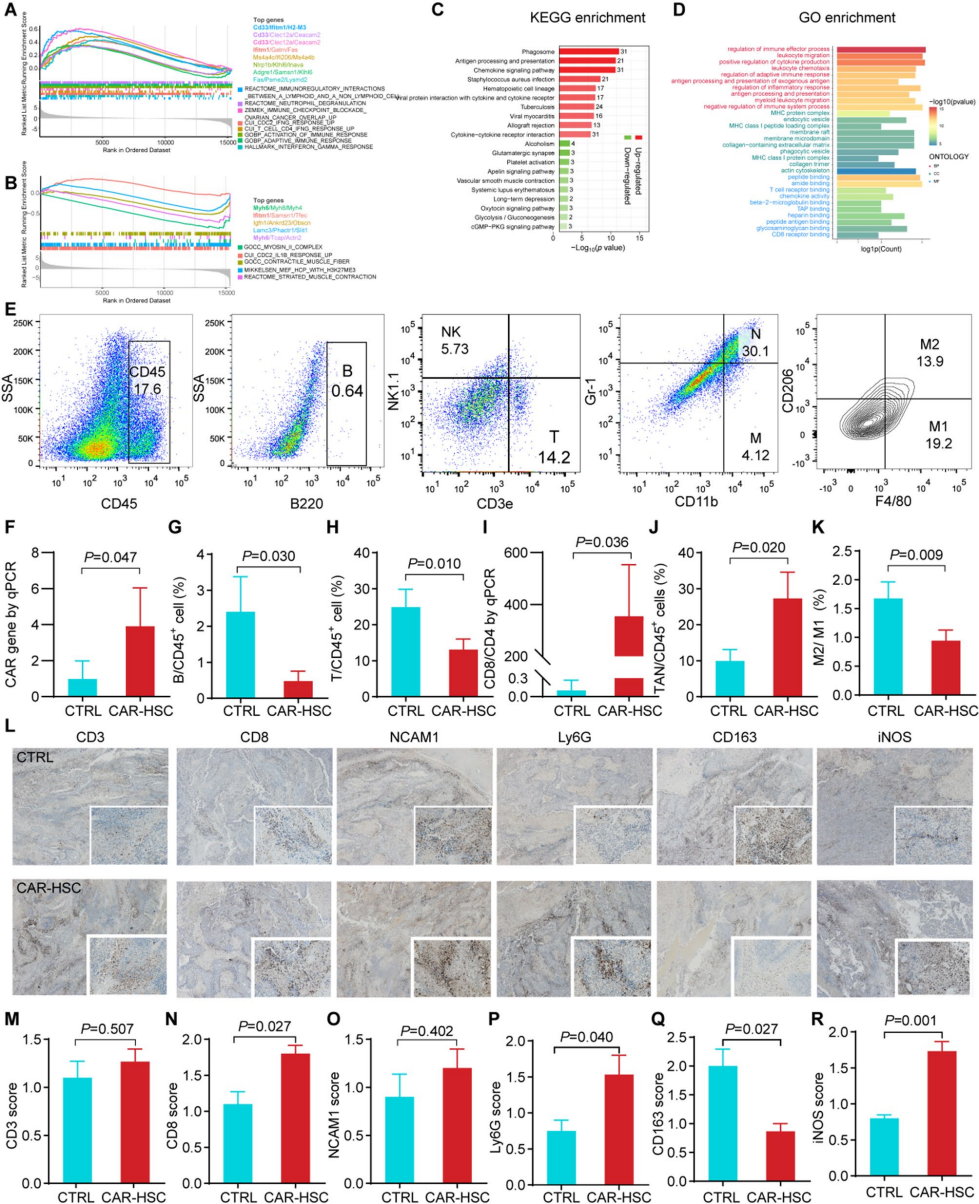
TME alteration after CAR-HSCs therapy. **A**–**B** GSEA analysis in the TME CAR-HSCs or HSCs transplantation. **C** KEEG pathway enrichment analysis in the TME of CAR-HSCs or HSCs transplantation. **D** GO enrichment analysis in the TME of CAR-HSCs or HSCs transplantation. **E** TME cell constituents by flow cytometry. **F** CAR-mRNA expression in TME by RT-PCR (*n* = 4). **G**–**H** Proportion of B cells and T cells in TME by flow cytometry (*n* = 4). **I** CD8/CD4 mRNA in TME by RT-qPCR (*n* = 4). **J**–**K** Proportion of TANs and M2/M1 ratio of TAMs in TME (*n* = 4). **L** IHC of TME. **M**–**O** Scores of Total CD3^+^ T cell, CD8^+^ T, NCAM1^+^ NK cell, Ly6G^+^ TANs, CD163^+^ M2 cells, iNOS^+^ M1 cells in TME (*n* = 4)

We further investigated the TME on day 20 post-transplantation. RNA-sequencing revealed the differentially expressed genes (DEGs) between the CAR-HSC and CTRL groups (Fig. S6A-B). The key upregulated immune-related genes such as *Cd33* and Ifitm1. *Cd33*, typically expressed in myeloid immune cells, may reflect enhanced recruitment or activation of antigen-presenting cells within the TME. *Ifitm1*, an interferon-stimulated gene, plays a critical role in antiviral defense and immune cell activation, aligning with the observed enrichment of interferon-γ response pathways (Fig. 2A). Conversely, *Myh6*, *Myh8*, and *Myh4*-genes encoding muscle-related contractile proteins-were significantly downregulated, suggesting disruption of stromal or muscle-like structural components within the TME (Fig. 2B). These upregulated genes involved lipid metabolism-associated genes, intracellular enzymes, and transcriptional regulator factors. Kyoto Encyclopedia of Genes and Genomes pathway analysis showed that the most positively enriched pathways were related to phagosome, antigen processing and presentation, and the chemokine signaling pathway (Fig. 2C). Gene Ontology enrichment analysis revealed that the main DEGs were involved in “regulation of immune effector process,” “leukocyte migration,” and “positive regulation of cytokine production” (Fig. 2D).

Changes in the immune cells in the TME were analyzed (Fig. 2E). The CAR gene was significantly higher in tumors in the CAR-HSC group than in the CTRL group (*P* = 0.047) (Fig. 2F). The CAR-HSC group showed lower proportions of B cells (*P* = 0.030) and T cells (*P* = 0.010) in the TME than the CTRL group (Fig. 2G-H). However, CD8/CD4 mRNA levels in the TME were significantly higher in the CAR-HSC group than in the HSC group (*P* = 0.036) (Fig. 2I). The CAR-HSC group showed a higher proportion of tumor-associated neutrophils (TANs) than the CTRL group (*P* = 0.020) (Fig. 2J). The tumor-associated macrophages (TAMs) subgroups in the CAR-HSC group had lower M2/M1 ratios (*P* = 0.009) (Fig. 2K). There was no significant difference in other immune cells between the two groups (Fig. S5).

The TME immunohistological analysis results (Fig. 2L) were consistent with the flow cytometry results. The CD3^+^ T cell score (Fig. 2M) was not statistically different between the two groups. However, the CAR-HSC group showed a higher CD8^+^ T cell score than the CTRL group (*P* = 0.027) (Fig. 2N). The NCAM1^+^ NK cell score was not statistically different between the two groups (Fig. 2O). The CAR-HSC group showed a higher Ly6G^+^ TAN score than the CTRL group (*P* = 0.040) (Fig. 2P). Regarding TAMs, the CAR-HSC group showed a lower number of CD163^+^ M2 cells (*P* = 0.027) and a higher inducible nitric oxide synthase (iNOS^+^) M1 cell score (*P* = 0.001) than the CTRL group (Fig. 2Q-R). These results indicate that CAR-HSCs transplantation reshaped the immunosuppressive TME to an immunologically active TME.

CAR-HSCs transplantation added a graft-*versus*-tumor effect to ASCT with no risk of graft-*versus*-host disease [16]. CAR-HSCs differentiated to CAR-T, CAR-NK, CAR-M, and CAR-neutrophils to kill tumor cells and reshaped the immunosuppressive TME to immunologically active TME [17]. Transcriptional alterations reveal that CAR-HSCs not only activate immune responses but also dismantle the physical and epigenetic barriers of an immunosuppressive TME, thereby facilitating immune cell infiltration and function. Moreover, multiple CAR-expressing cells homed to tissue and circulated in the peripheral blood, providing immune surveillance to prevent tumor relapse. Therefore, CAR-HSCs could potentially be used in the future for treating solid tumors, such as esophageal cancer and colorectal cancer [18, 19]. To enhance the safety of HSC genetic modification, we will incorporate a suicide gene into the gene transfer vector, which could allow selective elimination of modified cells in the event of toxicity.

In summary, this study provides a new potential therapy using CAR-HSCs transplantation, employing multiple CAR-expressing immune cells to kill tumor cells, reshaped the TME. Thus, CAR-HSCs transplantation might be a promising therapy for r/r LBCL in clinical practice.

## Limitations

The immune competent murine ASCT model was used, and the human CD19 antigen as an exogenous protein which may be attacked by murine immune cells and influence the efficacy of CAR-HSC. Furthermore, the long-term safety and efficacy of CAR-HSC have not been explored, because of the difficulty for the human CD19-bearing cell line to form tumors in immune competent mice.

## Supplementary Information

Below is the link to the electronic supplementary material.

Supplementary Material 1

## Data Availability

No datasets were generated or analysed during the current study.

## Abbreviations

LBCL: Large B-cell lymphoma
r/r: Relapsed or refractory
ASCT: Autologous hematopoietic stem cell transplantation
CAR-T: Chimeric antigen receptor T cell
TME: Tumor microenvironment
CAR-M: CAR-macrophage
HSCs: Hematopoietic stem cells
CAR-HSCs: CAR-modified HSCs
DEGs: Differentially expressed genes
TANs: Tumor-associated neutrophils
TAMs: Tumor-associated macrophages
