# Supplementary material for: Chimeric antigen receptor modified hematopoietic stem cells (CAR-HSCs) arm all immune forces for anti-tumor in mice
Source: Exp Hematol Oncol. 2025 Oct 22;14:124. doi: 10.1186/s40164-025-00715-7 (PMC12548230; doi:10.1186/s40164-025-00715-7)

**Chimeric antigen receptor modified hematopoietic stem cells (CAR-HSCs) arm all immune forces for anti-lymphoma in mice**

## **Supplementary Materials and Methods:**

### Mice and cell lines

C57/BL6J mice (B6-CD45.1 and B6-CD45.2) were obtained from the Jackson Laboratory and kept in a specific pathogen free (SPF) animal facility. All animal experiments were approved by the Committee on Ethics of Biomedicine Research of the Naval Medical University, Shanghai, China. Mice were sacrificed when tumor growth exceeded 10% of body weight, or tumor diameter exceeded 20 mm, or grew to the point of ulceration, causing infection or necrosis. Mouse melanoma B16 cell line was purchased from ATCC. Human CD19 protein and luciferase were overexpressed on B16 cell line, which were called B16-CD19-Luc, since B16 cell line is characterized by low immunogenicity for immune-competent mice. B16 and B16-CD19-Luc were cultured in 89% RPIM1640 complete medium containing 10% fetal bovine serum (FBS) and 1% antibiotics at 37 ℃ in a humidified atmosphere under 5% CO_2_.

### Vector manufacture and HSC transduction

As regards the structure of CAR, FMC63 single-chain fragment variable (scFv) followed by the CD8 transmembrane domain 4-1BB, and CD3ζ signaling domain were cloned into a lentiviral vector. HSC were isolated from the bone marrow of C57/BL6 mice using the EasySep™ Mouse Hematopoietic Progenitor Cell Isolation Kit (STEMCELL, no. 19856), and isolated HSCs were cultured in StemSpan™ Serum-Free Expansion Medium (STEMCELL, no. 09600) supplemented with 100 ng/ml mSCF (PEPRO TECH, no. 250-03), 100 ng/ml mFLT3L (R&D system, no. 427-FL-025), and 100 ng/ml mTPO (PEPRO TECH, no. 315-14). HSCs were cultured into 24-well plate at a concentration of 1×10^6^/ml. The CAR lentiviral vectors and polybrene (3 µg/ml) were added to the cells. Blank vector and polybrene (3 µg/ml) were transfected into the control (CTRL) group. The multiplicity of infection was 10. Cells were centrifuged at 1200 g for 2 hours at 32 ℃ and incubated at 37 ℃ in a humidified atmosphere under 5% CO_2_ for 24 hours.

### Colony-forming unit (CFU) assay

HSC or CAR-HSC was washed with Iscove’s MDM medium containing 2% FBS, counted and their density was adjusted to 1×10^4^/ml. A hundred µl cell suspension was mixed with 900 µl methylcellulose-based medium MethoCult™ GF M3434 (STEMCELL, no. 03434) and thoroughly vortexed. Cells were kept at 4 ℃ for 10 min to remove bubbles. Cells were seeded into a 24-well plate with a syringe and blunt-end needle and incubated at 37 ℃ and 5% CO_2_ for 10 days. The CFU of multi-potential neutrophil, erythroid, macrophage, megakaryocyte progenitor cells (CFU-GEMM), granulocyte-macrophage progenitor cells (CFU-GM), granulocyte (CFU-G), macrophage progenitor cells (CFU-M) and erythroid progenitor cells (BFU-E) were counted under an inverted microscope.

### ASCT and lymphoma mouse model

As regards the ASCT model, the B6-CD45.1 mice were used as donor mice and the B6-CD45.2 mice as the recipient mice. The donor mice were sacrificed, the long bones were collected, and the bone marrow cells were washed with ice-cold PBS and passed through 70 µm strainer. Cells were counted and their density was adjusted to 2×10^8^/ml for HSC purification using EasySep™ Mouse Hematopoietic Progenitor Cell Isolation Kit (STEMCELL, no. 19856) following the manufacturer’s protocol. The recipient mice (8 weeks, female) were irradiated with ^137^Cs at a lethal dose of 8 Gy. A total of 1×10^6^ isolated bone marrow cells HSC or CAR-HSC without isolation were injected into each mouse by tail-vein injection at 24 h after irradiation. Ciprofloxacin (1 g/L) was added to drinking water for 2 weeks from the day of irradiation. Chimeric rate (CD45.1/CD45) was assessed at 4 weeks after transplantation using flow cytometry.

As regards the lymphoma preclinical model, 2×10^5^ B16-CD19-Luc cells were subcutaneously injected into the inguinal region at 7 days after HSC transplantation, when the immune cells were at the lowest level. Tumor growth was detected at 6 days after inoculation by bioluminescence imaging (BLI).

### Antibodies and flow cytometry

Flow cytometry was performed with BD Aria Ⅱ and data were analyzed by FlowJo software (TreeStar, version 10.07). CAR detection was performed using fluorochrome-conjugated antibody (HuaDao biopharm). HSC phenotypes were detected using anti-Lineage (eBioscience, 887772-72), anti-CD117 (BD pharm, 553356) and anti-Sca-1 (BD pharm, 558162). Chimeric rate was assessed using anti-CD45.1 (BD pharm, 560579) and anti-CD45.2 (BD pharm, 561874). Peripheral blood cells were labeled with anti-CD45 (BD Horizon, 563891), anti-B220 (BD pharm, 553092), anti-CD3e (BD pharm, 557596), anti-NK1.1 (BD pharm, 552878), anti-CD11b (BD pharm, 562605) anti-Gr-1 (BD pharm, 553128), anti-F4/80 (BD Optibuild, 746070), and anti-CD206 (Thermo, 25-2061-82).

### Immunohistochemistry (IHC)

Mouse tissues were dissected, fixed with 10% formalin for 24 hours and embedded in paraffin. They were cut into 4 µm-thick sections for H&E staining and IHC. The primary antibodies used were the following: anti-CD3 (Servicebio, GB111337), anti-CD8 (Servicebio, GB13429), anti-NCAM1 (Servicebio, GB112671), anti-LY6G (Servicebio, GB11229), anti-CD163 (Servicebio, GB13340), and anti-iNOS (Servicebio, GB11119). The secondary antibody was HRP-labeled goat anti-rabbit IgG. The Positive Cell Proportion Scoring Method is applied for TME IHC evaluation, where specimens were graded 0-3 based on the percentage of stained positive cells:0 point (0%), 1 point (1-25%), 2 point (26-50%), 3 point (>50%). We count the number of positive cells in five randomly selected fields of view were counted for the percentage of positive cells. The tissue score is calculated as the average of these five scores.

### Luminex liquid suspension chip detection

Mice serum was centrifuged at 1000 g for 10 min and diluted 1:3 with sample diluent. Cytokine detection was performed using Bio-Plex Pro Mouse Cytokine 23-Plex Immunoassay Bio-Plex Pro Mouse Cytokine Grp (#M60009RDPD) according to the manufacturer’s protocol and analyzed using Bio-Plex 200 System (Austin, TX, USA). The cytokine assay was performed by Wayen Biotechnologies Shanghai, Inc.

### RNA sequencing (RNA-seq)

Total RNA was extracted using miRNeasy Micro Kit (Qiagen, 217084), and RNA quantity and integrity were assessed using Agilent 4200 TapeStation (Agilent Technologies, Santa Clara, CA, USA). RNA library preparation was performed using VAHTS Universal V6 RNA-seq Library Prep Kit for Illumina (Vazyme, NR604,) according to the manufacturer's instructions. The sequencing was performed on an Illumina NovaSeq platform (Illumina, San Diego, CA, USA) and analyzed at Shanghai Biochip Co., Ltd., Shanghai, China. The fasted files of sequencing were mapped to the mouse genome GRCm39.104 and read counts of the genes were quantified by htseq-count (version 0.13.5). Gene Ontology (GO) and Kyoto Encyclopedia of Genes and Genomes (KEGG) analyses were performed using R bioconductor.

### Statistical analysis

Statistical analysis was performed using SPSS (version 26.0) and GraphPad (version 8.0.1). The comparison of quantitative data between two groups was performed using two-tailed Student’s *t*-test, while multiple groups were compared using ANOVA. The comparison of CFUs among three groups was performed using R×C χ² test and adjusted with bonferroni method. Mice survival was assessed by Kaplan-Meier analysis and log‐rank test. A value of *P* < 0.05 was considered statistically significant.

**Supplementary Figures and Figure Legends:**

**Fig. S1.**
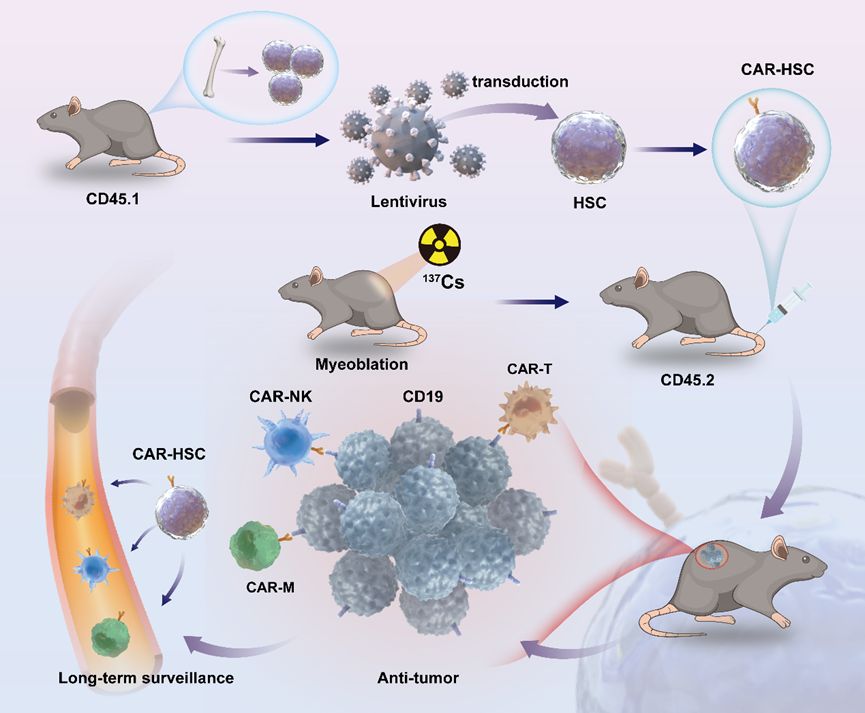
 **CAR-HSC differentiation and jointing effects of CAR-expressing immune cells.**

**
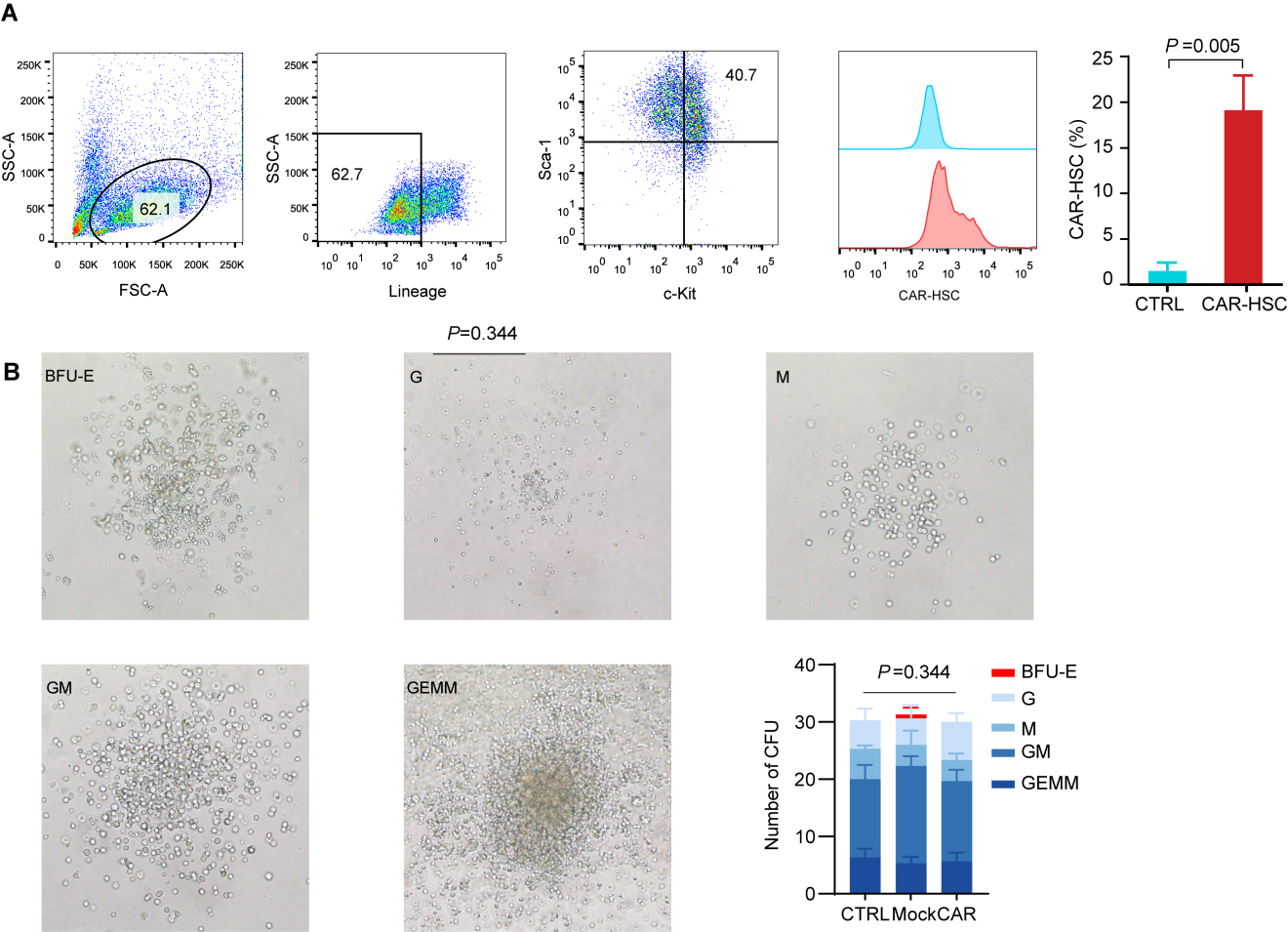
Fig. S2. Hematopoietic stem cells (HSCs) transduction with lentiviral vector containing the CD19 chimeric antigen receptor (CAR) gene without compromising its capacity of differentiation.** (A) CAR expression in HSCs (Lineage^-^Sca-1^+^C-kit^+^) by flow cytometry. (B) The Colony-forming unit (CFU) assay of unmodified HSCs, Mock-transduced HSC and CAR-HSC (100× magnification) (n=3).

**
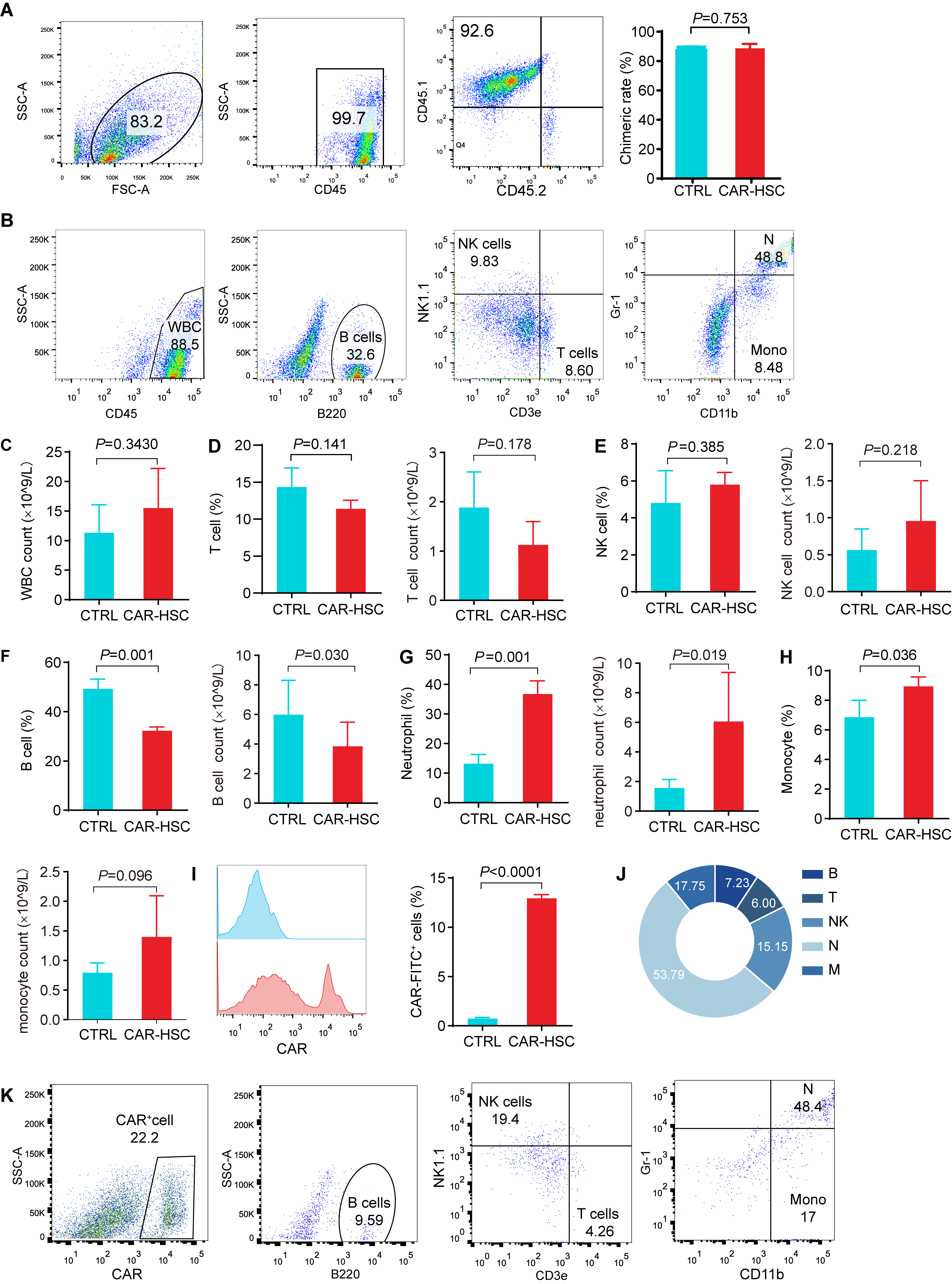
Fig. S3. CAR-HSCs hematopoietic reconstitution *in vivo*.** (A) Chimeric rate of mice transplanted with the CAR-HSCs or HSC (CTRL) by flow cytometry (n=4).(B) Different immune cells in the peripheral blood by flow cytometry. (C-H) The total white blood cell (WBC), proportion and absolute cell counts of peripheral T cell, NK cell, B cell, neutrophil and monocyte in the CAR-HSC and CTRL groups (n=4). (I) CAR expression in peripheral blood cells by flow cytometry (n=4). (J-k) Constituent of CAR-expressing cells in peripheral immune cells (n=4).

##
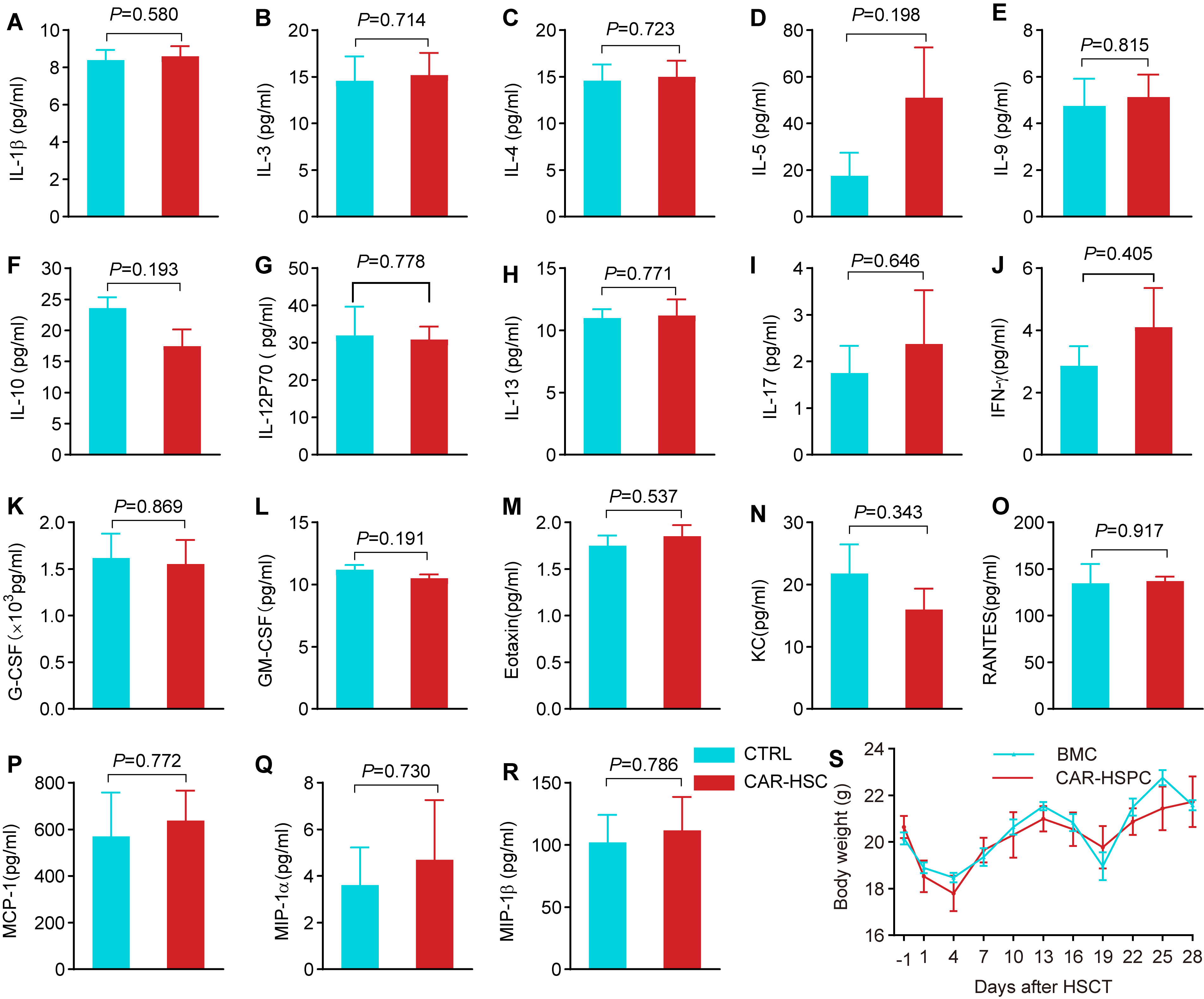
Fig. S4. Cytokine release syndrome evaluation. A-R.Serum cytokines on day 28 post-transplantation in CAR-HSC and HSC groups (n=3). S. The body weight of mice in CAR-HSC and HSC group (n=5).

##
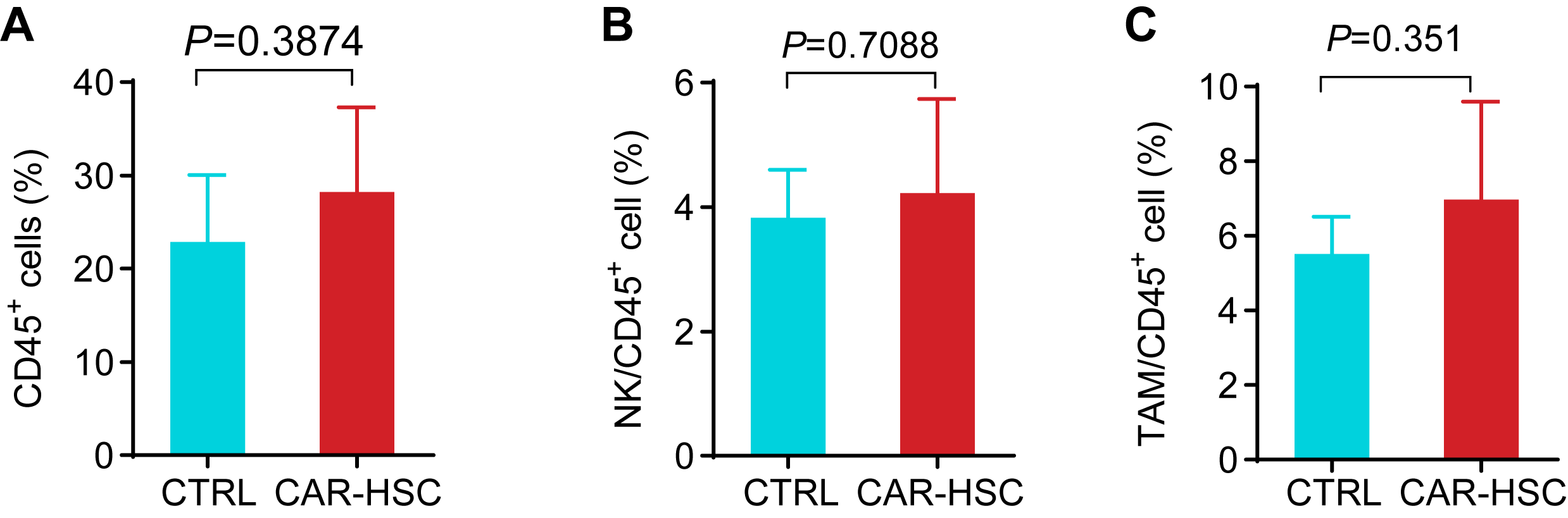
Fig. S5. Total immune cells, NK cells and TAM in TME. (A) Total immune cells. (B) NK cells. (C) Tumor-associated macrophages (TAMs) (n=4).

## Fig. S6. RNA sequencing reveals differentially expressed genes (DEGs) of TME **after CAR-HSCs therapy**. (A) Heatmap of DEGs in the TME of CAR-HSCs or HSCs transplantation. (B) Volcano map of DEGs in the TME of CAR-HSCs or HSCs transplantation.
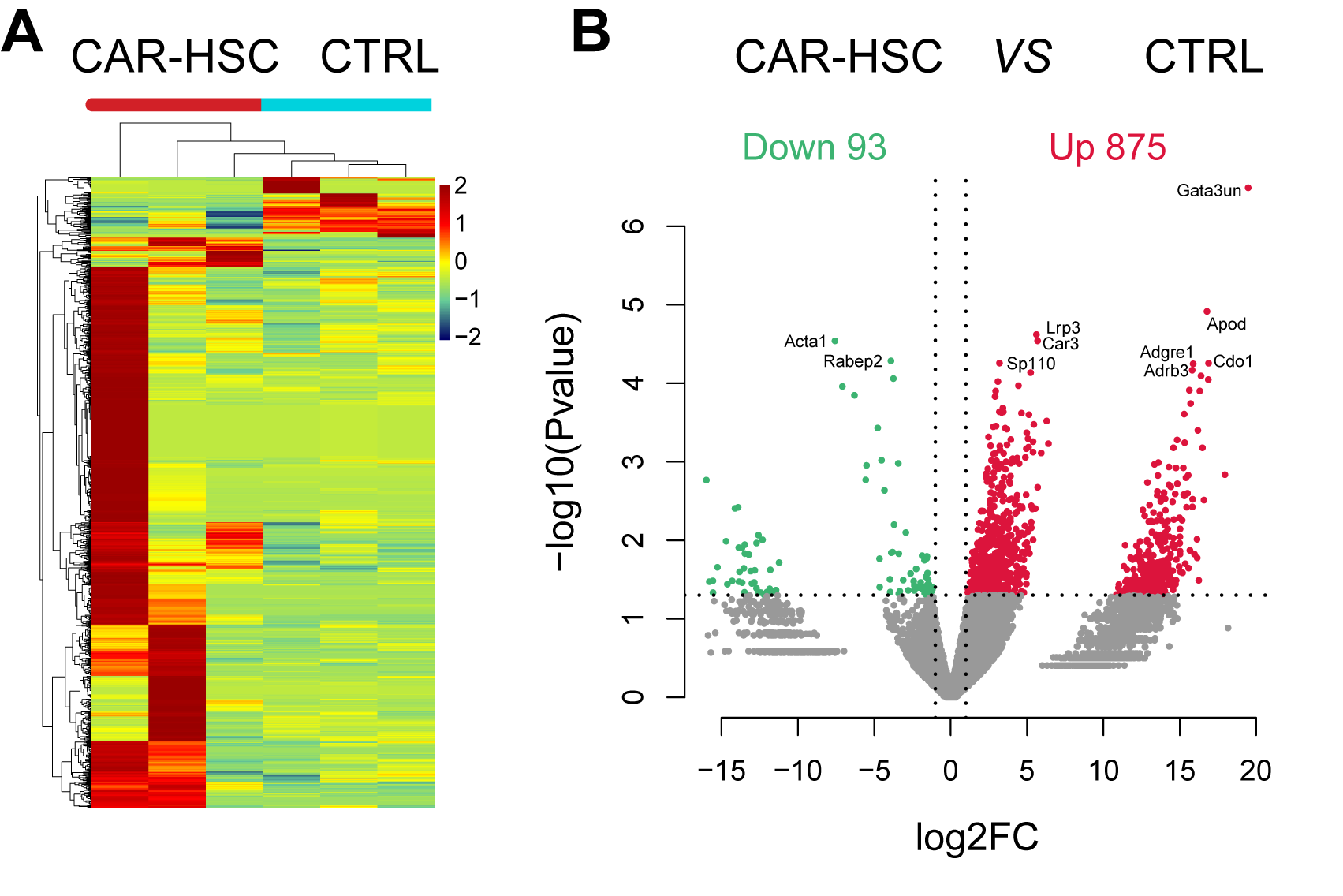

Supplement: Supplementary file 1 — Supplementary Material 1 [file 40164_2025_715_MOESM1_ESM.docx]
